# Supplementary material for: Distinctive alteration of presynaptic proteins in the outer molecular layer of the dentate gyrus in Alzheimer’s disease
Source: Brain Commun. 2021 May 13;3(2):fcab079. doi: 10.1093/braincomms/fcab079 (PMC8117432; doi:10.1093/braincomms/fcab079)
Supplement: fcab079_Supplementary_Data [file fcab079_supplementary_data.pdf]

Supplementary figures

Supplementary Figure 1

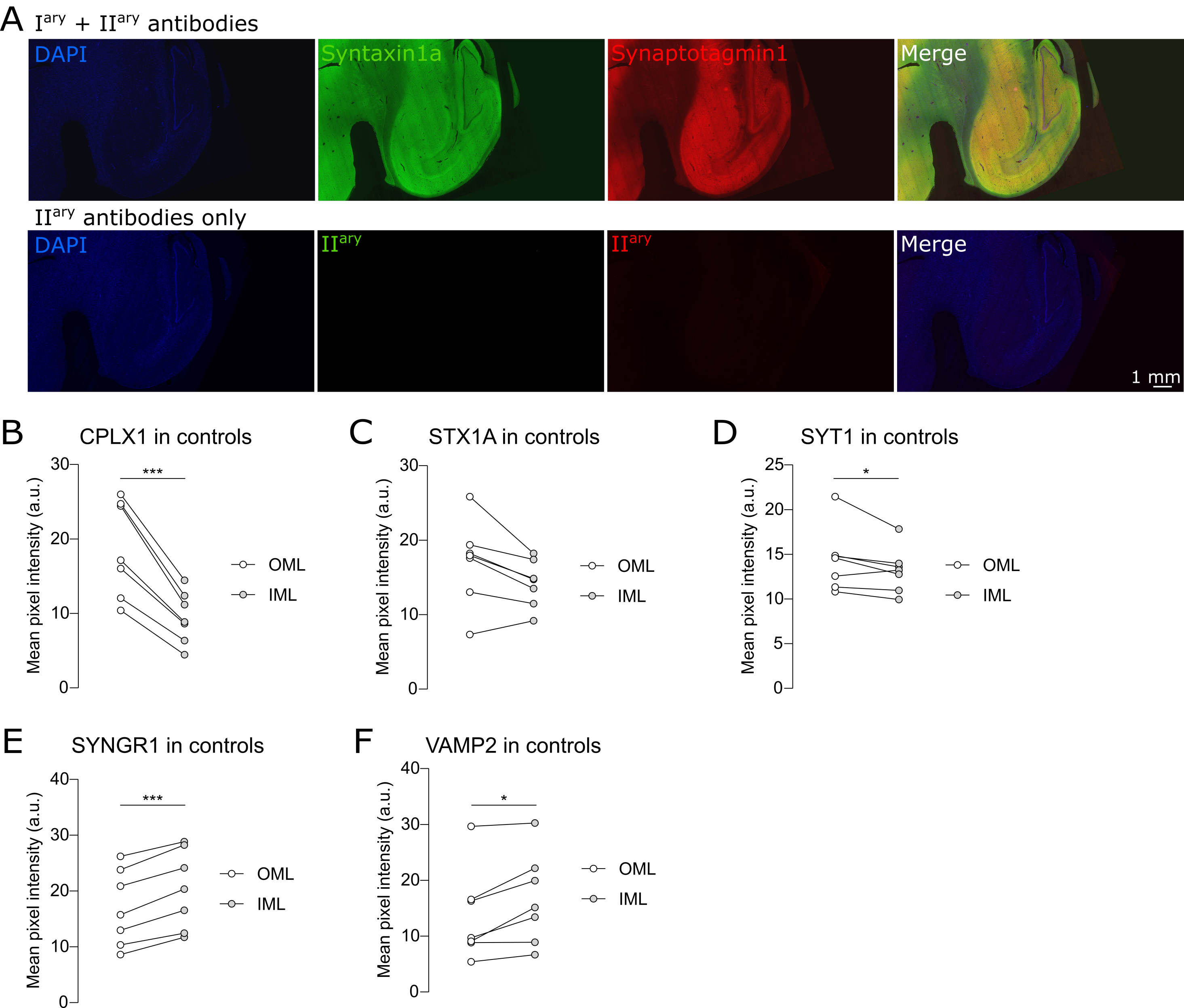

Supplementary Figure 1. Impaired OML/IML intensity ratio for presynaptic proteins (A) Controls of fluorescent immuno-labelling in human hippocampal sections. In the upper panels, the sections are stained for DAPI, syntaxin1a and Syt1. In the lower panels, only secondary antibodies were incubated in addition to DAPI. The fluorescent immuno-labelings are specific to the primary antibodies. (B-F) Before-after plot of the mean pixel intensity in controls of the fluorescent labellings in OML or IML respectively for CPLX1, STX1A, SYT1, SYNGR1, VAMP2. The pixel intensities were significantly higher in OML compared to IML by 99% for CPLX1 (B, OML: 18.7+/-6.4, IML: 9.5+/-3.5; p-value < 0.0001) and by 8% for SYT1 (D, OML: 14.3+/-3.6, IML: 13.2+/-2.5; p-value = 0.038) and were higher in IML by 17 % for SYNGR1 (E, OML: 16.9+/-6.8, IML: 20.3+/-7.1; p-value = 0.0003) and by 20% for VAMP2 (F, OML: 13.7+/-8.2, IML: 16.6+/-8.2; p-value = 0.016). Statistical descriptions are described as mean+/-S.D. p-values refer to ratio paired, two-tailed *t*-test.

Supplementary Figure 2

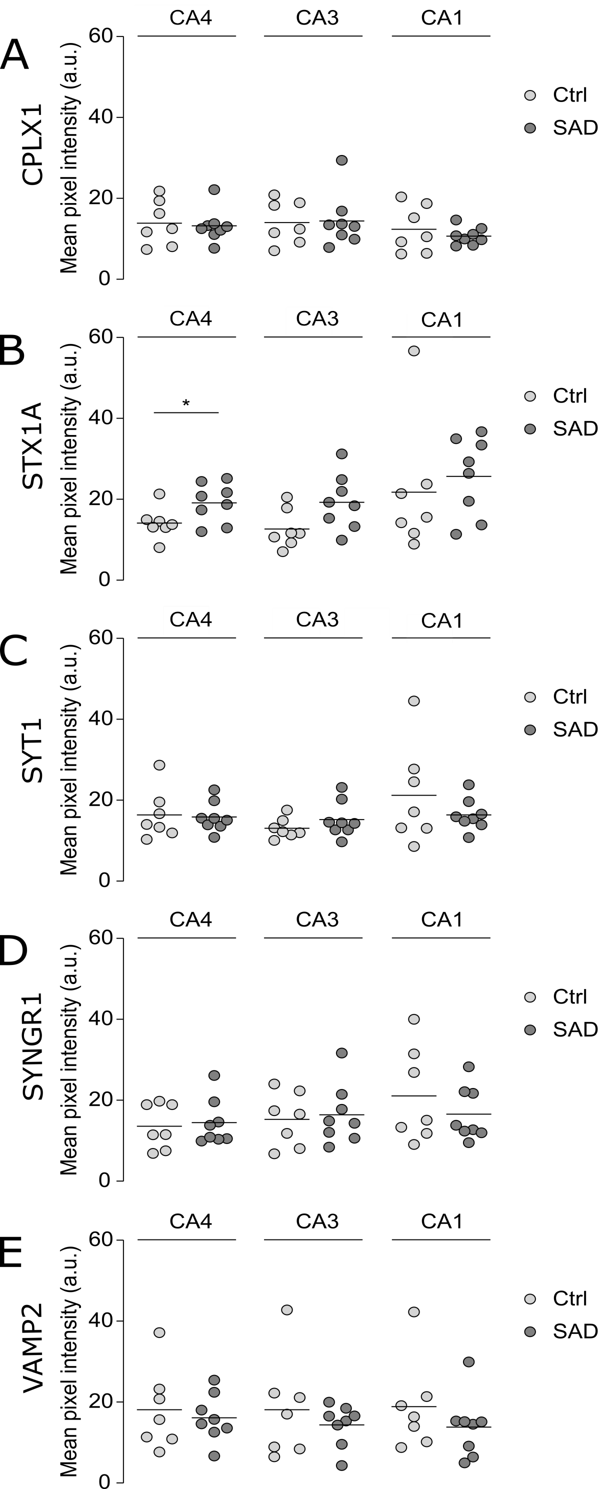

Supplementary Figure 2. Preserved level of presynaptic proteins in various neuronal layers of the hippocampus (A-E) Scatter plots of the mean pixel intensity of the fluorescent labellings in CA3, CA1 and CA4 respectively for CPLX1, STX1A, SYT1, SYNGR1, VAMP2. The fluorescence pixel intensities were not altered in these regions except for STX1A which was increased in AD CA4 (B, ctrl: 14.1+/-3.9, AD: 19.1+/-4.9; p = 0.0485). Moreover, a non-significant trends towards increased levels of STX1A were detected also in the CA3 neuronal layer (B, ctrl: 12.7+/-4.8, AD: 19.3+/-6,8; p-value = 0.0506).
